# Helicate versus Mesocate in Quadruple-Stranded Lanthanide Cages: A Computational Insight

**DOI:** 10.3390/ijms231810619

**Published:** 2022-09-13

**Authors:** Silvia Carlotto, Lidia Armelao, Marzio Rancan

**Affiliations:** 1 Department of Chemical Sciences (DiSC), University of Padova, Via F. Marzolo 1, 35131 Padova, Italy; 2 Institute of Condensed Matter Chemistry and Technologies for Energy (ICMATE), National Research Council (CNR), c/o Department of Chemical Sciences (DiSC), University of Padova, Via F. Marzolo 1, 35131 Padova, Italy; 3 Department of Chemical Sciences and Materials Technologies (DSCTM), National Research Council (CNR), Piazzale A. Moro 7, 00185 Roma, Italy

**Keywords:** helicate, mesocate, quadruple-stranded, lanthanides, DFT

## Abstract

To drive the synthesis of metallo-supramolecular assemblies (MSAs) and to fully exploit their functional properties, robust computational tools are crucial. The capability to model and to rationalize different parameters that can influence the outcome is mandatory. Here, we report a computational insight on the factors that can determine the relative stability of the supramolecular isomers helicate and mesocate in lanthanide-based quadruple-stranded assemblies. The considered MSAs have the general formula [Ln_2_L_4_]^2−^ and possess a cavity suitable to allocate guests. The analysis was focused on three different factors: the ligand rigidity and the steric hindrance, the presence of a guest inside the cavity, and the guest dimension. Three different quantum mechanical calculation set-ups (in *vacuum*, with the solvent, and with the solvent and the dispersion correction) were considered. Comparison between theoretical and experimental outcomes suggests that all calculations correctly estimated the most stable isomer, while the inclusion of the dispersion correction is mandatory to reproduce the geometrical parameters. General guidelines can be drawn: less rigid and less bulky is the ligand and less stable is the helicate, and the presence of a guest can strongly affect the isomerism leading to an inversion of the stability by increasing the guest size when the ligand is flexible.

## 1. Introduction

Self-assembly of discrete metallo-supramolecular architectures (MSAs) paves the way towards functional systems such as helicates, grids, wheels, knots, and cages that in the last three decades have shown increasing complexity [1,2,3]. MSAs display a wide range of applications spanning from magnetism to catalysis in confined cavities [4,5,6,7]. The self-assembly and properties of these systems are orchestrated by an array of components including the metal ions with different coordination geometries and numbers, different charge and spin, and ligands with distinct size, shape, and chemical properties such as spacer length, flexibility, binding groups, and steric hindrance. The effect of these main components is often coupled with more subtle parameters such as the solvent, counterions, and guest molecules.

Among possible MSAs, helicates are one of the longest-known systems. The term was coined by Lehn and co-workers [8] to describe an MSA where ligands are wrapped around metal ions with a helical twist. Single-, double-, triple-, quadruple-stranded, and circular helicates have been reported over time [9,10,11]. From the very beginning, helicates, due to their chiral supramolecular structure and because of their structural analogy with helical biomacromolecules (such as DNA and α-helices), have attracted much interest [12,13,14,15]. The chirality of helicates arises from the propeller-like coordination arrangement (Λ or Δ) of the ligand binding groups around the metal centers leading to helical structures (*M* or *P*). However, if the metal centers possess opposite-handedness, the final MSA is achiral and is called mesocate. Helicate and mesocate are hence supramolecular isomers chiral and achiral, respectively. Although these structures have been known for some decades [8,16,17], the factors that allow driving the formation of the helicate or mesocate are still not properly understood. Thus, it is difficult to find general rules able to predict the most stable isomer and its geometrical properties. The literature has reported many possible factors that determine the formation of one isomer with respect to the other, such as: (i) the metal ion dimensions [18,19]; (ii) the steric hindrance of the ligand [19] or (iii) the spacer length (odd–even rule) [20]. However, Dolphin [21] reported that the same ligand and metal can lead to the formation of both isomers, while Raymond [22] highlighted how the host–guest interaction (even with the solvent) can shift the equilibrium toward the otherwise less stable species. In this context, recently we reported a series of lanthanide [Ln_2_L_4_]^2−^ quadruple-stranded helicates that show interesting supramolecular properties such as ion exchange to produce heterometallic systems [23] and adaptive helicity reorganization due to a guest-to-host chirality transfer [24]. We also reported a DFT study for the helicity inversion and helicate–mesocate interconversion based on a Bailar twist, which demonstrated that the ligand scaffold nature (flexibility versus rigidity) plays a crucial role on the activation energy of the intramolecular helicate twisting mechanism [24]. Such lanthanide helicates, which display a unique combination of confined cavities, adaptive chirality, heteronuclear structures, and peculiar Ln luminescent properties, are particularly interesting for the development of chiro-optical probes for selective sensing via molecular recognition.

**Figure 1:**
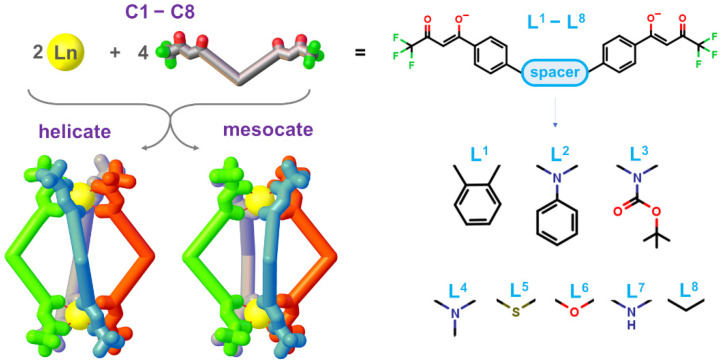
[Ln_2_L_4_]^2−^ cages in the helicate and mesocate forms and general formula of the bis-β-diketone ligands L^1^−L^8^ with the different considered spacers.

To drive the synthesis and functional properties of new MSAs through a new effective and feasible approach, a robust computational tool is crucial. The computational investigation allows the systematic study of various types of MSAs, by varying the flexibility, steric hindrance, and chemical properties of the organic ligands and connecting these characteristics with specific features. The rationalization of each MSA component will allow building of multifunctional coordination by introducing different ligands with different properties. Among MSAs, coordination-driven cage compounds have surely attracted much attention even for the development of new dedicated computational tools to design new metallo-cages and to model their behavior [25,26,27,28,29]. On the contrary, the computational literature on lanthanide-based helicates (triple- and quadruple-stranded) is quite poor. Recent studies reported Perdew–Burke–Ernzerhof (PBE) in *vacuum* calculations for empty quadruple-stranded helicates, with Ln(III) ions and aromatic β-diketones [30], and also, in our preview studies, the same functional with the inclusion of solvent and dispersion correction was used to reproduce the geometrical structure [23] and the isomer stability [24] of the lanthanide organic cages. In addition, molecular mechanic calculations (Sparkle/RM1 model) on quadruple-stranded dinuclear Eu(III) helicate with bis-β-diketone ligand were considered [31,32]. Hybrid functionals (B3LYP) with the inclusion of the solvent or dispersion are reported for empty double- and triple-stranded helicates, [33,34,35,36], hence for smaller systems with respect to the quadruple-stranded helicates. A study with a non-hybrid BP86 functional in *vacuum* is also presented for triple-stranded structures [37]. The common aspect of the theoretical current literature on triple- and quadruple-stranded cages is the lack of (i) a systematic investigation of the role of the accuracy of quantum mechanical calculations as the inclusion of solvent and/or dispersion on the agreement with experimental data, and (ii) the influence of the ligand properties, or (iii) the presence of a guest on geometrical parameters and on the stability of the different isomers (helicate and mesocate). In this study, we use quadruple-stranded [Ln_2_L_4_]^2−^ (Figure 1) systems to systematically investigate these blanks. Three different computational set-ups were considered: in *vacuum*, with the inclusion of the solvent (named solvent), and with the inclusion of the implicit solvent and the dispersion correction (named solvent-D). The cavity containing quadruple-stranded [Ln_2_L_4_]^2−^ systems allows evaluation of three main factors: (i) the ligand rigidity and steric hindrance; (ii) the presence of a guest inside the cavity; and (iii) the guest dimension. The MSAs analyzed with DFT calculations have the general formula [La_2_L^X^_4_]^2−^ for the host systems and {NR_4_⊂[La_2_L^X^_4_]}^−^ for host–guest systems. Systems obtained with eight different ligands, L^1^−L^8^, were analyzed. The ligands are bis-β-diketone with the same binding groups, but they display scaffolds with variable flexibility and steric hindrance (see Figure 1), while the NR_4_^+^ guests are tetraalkylammonium cations with increasing dimension (R = Me, Et, Pr, Bu). For clarity and concision, hereafter, the MSAs [La_2_L^1−8^_4_]^2−^ will be labeled as **C1**−**C8** depending on the used ligand. 

## 2. Results and Discussion

**Figure 2:**
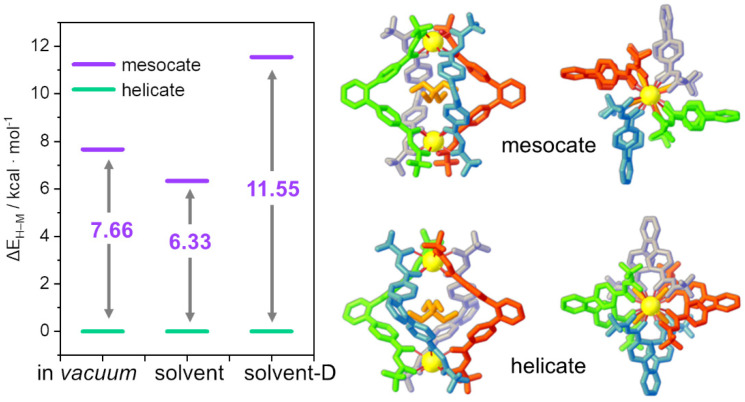
Energy difference between helicate and mesocate isomers (ΔE_H–M_, kcal/mol) for the test system NEt_4_⊂**C1**. Side and top views of the helicate (bottom) and mesocate (upper) isomers.

The computational set-up was implemented on the NEt_4_⊂**C1** cage bearing the most rigid ligand and hosting the cation NEt_4_^+^. La^3+^ was used as a metal center, because of its closed-shell electronic configuration—[Xe]4f^0^—that allows simplification of the level of theory used to opportunely describe the systems. This Ln-substitution was previously successfully applied in smaller complexes [38]. Three different numerical experiments were considered: (i) in *vacuum*, (ii) with the presence of an implicit solvent (acetonitrile, MeCN), and (iii) with the presence of the implicit solvent coupled to dispersion correction (solvent-D). Figure 2 shows that, for all methods, in agreement with experimental results [24], the helicate isomer is more stable than the mesocate isomer. For the in *vacuum* and solvent calculations, the stabilization of the helicate with respect to the mesocate (ΔE_H–M_) is very similar (7.66 and 6.33 kcal/mol, respectively), while the inclusion of the dispersion sensitively enhanced the helicate stability (11.55 kcal/mol). These outcomes show that all methods correctly reproduce the experimental data on helicate stability. 

**Figure 3:**
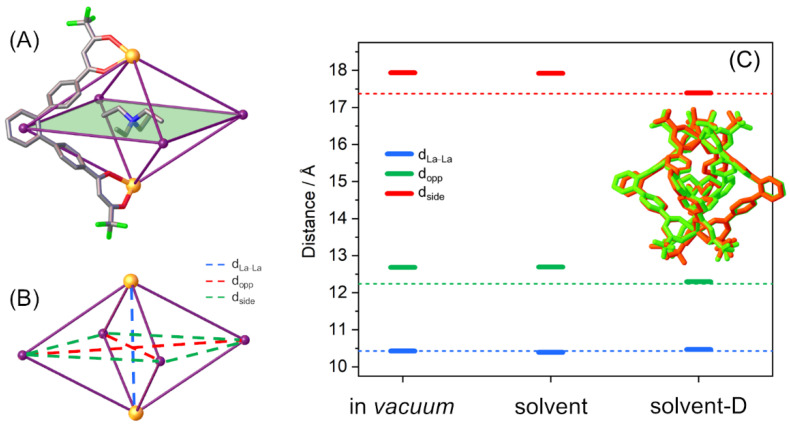
(**A**,**B**) Schematization of the cages as a pseudo-octahedron: (**A**) NEt_4_⊂**C1** cage with the equatorial plane highlighted in green, where three ligands were omitted to highlight the pseudo-octahedral geometry and (**B**) octahedral structure. The dashed lines indicate the three different distances: along the axial direction (d_La–La_, blue) and on the equatorial plane (d_opp_, red, and d_side_, green). (**C**) The solid bars represent the calculated values for the different distances along the axial direction (d_La–La_, blue) and on the equatorial plane (d_opp_, red, and d_side_, green). The dashed lines indicate the SCXRD data values [24]. Distances are in Å. In the inset, the overlap between the SCXRD structure (orange) and the geometry optimized with solvent and dispersion correction (green).

However, even if all methods are almost equivalent to predicting the most stable isomer, their performances are strongly different when comparing the capability to reproduce the geometrical and structural parameters of the NEt_4_⊂**C1** cage. In order to determine and quantify the methods’ accuracy, cage description was simplified to a pseudo-octahedron (Figure 3A), where the six vertices are the two La^3+^ ions and the four centroids of the ligand central scaffold. Three different distances were defined (Figure 3B): (i) one along the axial direction connecting the two La^3+^ ions (d_La–La_, blue dashed line); (ii) two on the equatorial plane, i.e., the distances connecting two opposite ligands’ spacer centroids (d_opp_, red dashed lines) and the distances of the equatorial side (d_side_, green dashed lines). For the equatorial plane distances (d_opp_ and d_side_), the average values were considered.

Calculated distances for NEt_4_⊂**C1** were compared to the SCXRD experimental structure [24] (see Table 1). The in *vacuum* and solvent calculations show similar percentage errors (about 4%), which are drastically reduced (below 0.5%) with the inclusion of dispersion correction for the equatorial distances (d_opp_ and d_side_). On the other hand, for d_La–La_ distance, the variation is negligible and there is good accordance with the experimental value with all three calculation set-ups. These trends are clearly highlighted in Figure 3C, where the different distances obtained with all three set-ups are compared with the SCXRD experimental values (dotted lines). The overlap between SCXRD experimental and calculated optimized (solvent-D) structures is reported as the inset of Figure 3C. 

After validation of the computational set-up, it was applied to different cages by systematically changing the ligand and the guest. Indeed, in the studied systems, three factors can mainly influence the helicate–mesocate equilibrium (ΔE_H–M_): (i) the ligand nature (rigidity and steric hindrance) (ii) the presence of a guest inside the cavity; and (iii) the guest dimension. Firstly, the focus was on the performance of the different computational set-ups on stability and structural properties, and secondly, on how ligand and guest characteristics can affect the stabilization of one isomer with respect to the other and the cage structure. 

### 2.1. The Influence of the Ligand on the Cage

The nature of the ligand in the cage may influence both the stability of the helicate/mesocate isomers and the cage structure. A series of eight cages bearing eight different ligands, as reported in Figure 1, has been studied. The host–guest system NEt_4_⊂**C** cages were considered due to the possibility of a direct comparison with SCXRD data for some systems (NEt_4_⊂**C1** [24], NEt_4_⊂**C3** [23], NEt_4_⊂**C8** [24]), while the choice of the other five ligands is mainly due to their different rigidity and steric hindrance. Indeed, from **C1** to **C2**, **C3** and **C4**, and then **C8**, there is a progressive increment in the flexibility of the ligand. The four N cages (**C2**, **C3**, **C4**, and **C7**) should have quite similar flexibility because the spacer is always the nitrogen atom, but an incremental steric hindrance, from **C2** to **C4** and then **C7**, is observed. With the presence of this quartet, the role of the steric hindrance parameters can also be investigated under almost constant flexibility conditions. Moreover, to investigate in a very fine way the role of rigidity/steric hindrance in helicate stabilization, the spacer is variated from C to N and then O and S atoms, where zero, one, and two lone pairs are present for **C8**, **C7**, and **C6**/**C5** cages, respectively. 

#### 2.1.1. The Ligand Role in the Supramolecular Helicate/Mesocate Isomerism

From an experimental point of view, the rigidity of the ligands seems to be the most influential parameter on the relative stability of the two isomers. NEt_4_⊂**C1** and NEt_4_⊂**C3** cages crystallize as the helicate isomer [23,24], while the NEt_4_⊂**C8** crystallizes as the mesocate [24]. The literature has also reported a stable helicate isomer for the **C4** [39] and **C5** [40] cages, even if the guests are different (diethyl ether for **C4** and chloroform for **C5**). As far as the other ligands are concerned (**C2**, **C6**, and **C7** cages), to the best of our knowledge, no experimental SCXRD structures are available, even if **C2** has been described as an empty helicate [31]. Despite this, we included these systems in the calculations as well to test the influence of the chemical nature of the spacer.

**Figure 4:**
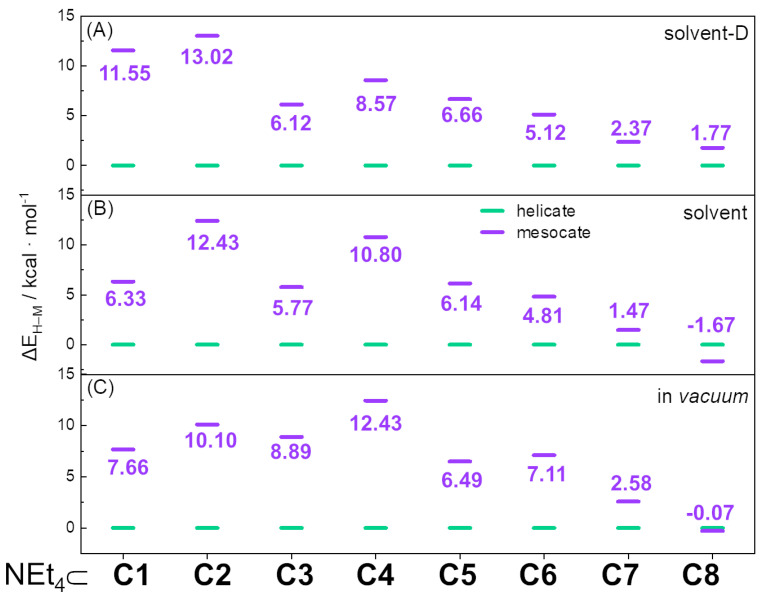
Energy differences ΔE_H–M_ (kcal/mol) between helicate (green) and mesocate (violet) isomers for in-solvent-D (**A**), in-solvent (**B**), and in *vacuum* (**C**) calculations. The energy of the helicate is considered 0.

The cage with the most rigid and highest steric hindrance ligand, NEt_4_⊂**C1**, is a helicate isomer from both experimental (SCXRD) and theoretical points of view (see Figure 4 and Table 1). This system and relative calculations have been discussed in detail in the previous section; hence, the attention will be focused on the other ligands. On the contrary, for the cage with the most flexible and lower steric hindrance ligand, NEt_4_⊂**C8**, where SCXRD data show a mesocate [24], only the in-solvent calculation correctly reproduces this evidence (Table 1). However, the calculated energy differences between the two isomers for all numerical experiments are tiny, below 2 kcal/mol (see Figure 4 and Table 1). Hence, it is very difficult to definitively discriminate between helicate and mesocate isomers. Even if for this cage the relative stabilities are almost negligible, the difference compared to the NEt_4_⊂**C1** cage is net: decreasing the rigidity and steric hindrance, there is a clear increment of the stability of the mesocate isomer (see Figure 4), in agreement with experimental results. 

The behavior of the other ligands can be explained as intermediate situations between **C1** and **C8** cages. In particular, the quartet NEt_4_⊂**C2**, NEt_4_⊂**C3**, NEt_4_⊂**C4**, and NEt_4_⊂**C7** cages have a decrescent rigidity and steric hindrance. SCXRD data for NEt_4_⊂**C3** [23] report a helicate isomer and all calculations, confirming experimental outcomes (see Figure 4 and Table 1). Calculations for **C2**, **C4**, and **C7** cages found the helicate isomer as the most stable isomer, even if the stability of the helicate isomer shows a clear decrescent trend. Another interesting trend can be drawn by considering the quartet NEt_4_⊂**C8**, NEt_4_⊂**C7**, NEt_4_⊂**C6**, and NEt_4_⊂**C5** cages. The first cage has no lone pair, the second has one, and the third and the fourth have two. In this case, the higher the number of lone pairs (i.e., steric hindrance), the higher the stability of the helicate isomer (see Figure 4 and Table 2).

The overall comparison between all calculations for all cages shows two clear correlations: the energy difference between helicate and mesocate tends to decrease with the increment of the flexibility of the ligand and with the decrement of the steric hindrance. The values for the relative stability of the isomers slightly change in *vacuum*, in-solvent, and in-solvent-D calculations, but this trend is perfectly reproduced in all numerical experiments (see Figure 4), even taking into account that an energy variation below 2 kcal/mol is not significant. 

#### 2.1.2. The Ligand Role on the Cage Structure

**Figure 5:**
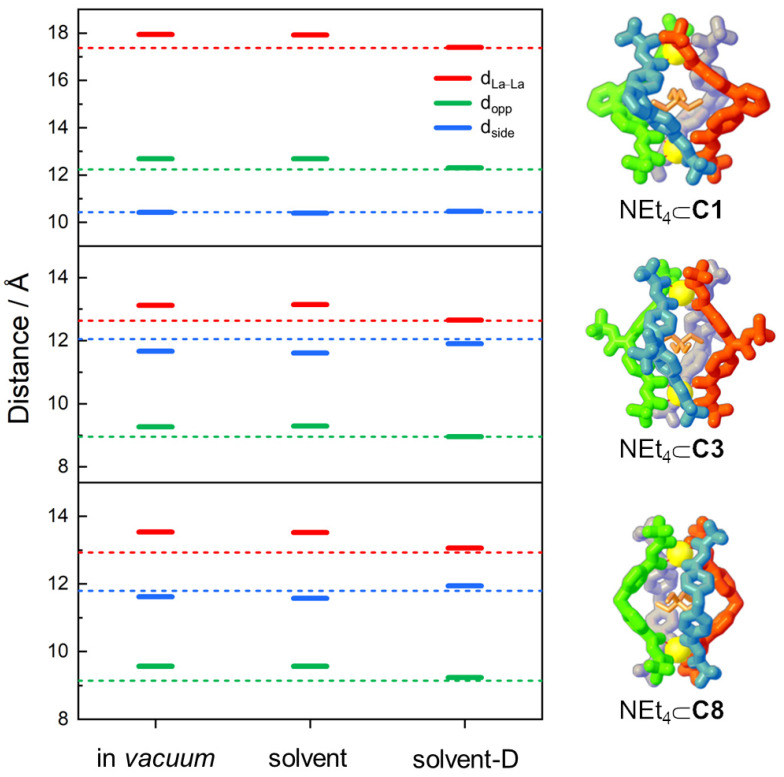
The solid bars represent the calculated values for the different distances along the axial direction (d_La–La_, blue) and on the equatorial plane (d_opp_, red, and d_side_, green), for the most stable structures. The dashed lines indicate the SCXRD data values. Distances are in Å.

While in *vacuum*, in-solvent, and in-solvent-D calculations show very close performance to reproduce the relative stability of one supramolecular isomer, their different abilities are better highlighted in the cage structure lengths (see Figure 5), as already mentioned for the L^1^ cage. 

The experimental and calculated geometrical parameters for **C1**, **C3**, and **C8** cages can be directly compared because the SCXRD data are recorded with the same guest, the NEt_4_^+^ [23,24]. Thus, the presence of the same guest induces us to attribute the structural variations only to the different ligands. Geometrical parameter trends for the **C1**, **C3**, and **C8** cages are similar (see Figure 5). In particular: (i) in *vacuum* and solvent calculations have similar values, with errors of around 4% with respect to experimental distances, while (ii) the solvent-D calculation shows a higher agreement with SCXRD data, with average errors of around 1% (see Table 1). Calculated geometrical parameter trends are equal for all cages: with the inclusion of solvent and dispersion correction, the d_side_ and d_opp_ values are compressed, while the d_La–La_ values are enlarged (see Table 1). It is interesting to highlight that the average errors on the more accurate calculation (solvent-D) increase progressively from **C1** (0.3%) and **C3** (0.5%) to **C8** (1.1%). This trend could be due to the increment of the flexibility of the ligand: a rigid ligand has fewer degrees of freedom, hence fewer stable conformations. When extending the analysis of the calculated geometrical parameters to all other cages, the abovementioned trends remain common: (i) the d_side_ and d_opp_ values are compressed, while (ii) the d_La–La_ values are enlarged (see Table S1 of the Supplementary Materials). 

### 2.2. The Influence of the Guest on Cage

The influence of the guest on supramolecular isomerism (helicate/mesocate stability) and on geometrical parameters was analyzed on different **C1** and **C8** cages, namely, the empty cage, i.e., the hosts **C1** or **C8**, and on the host–guest systems NR_4_⊂**C**, where R = Me, Et, Pr, Bu. The choice of the guests, a series of tetraalkylammonium ions, was guided by experimental evidence (SCXRD), which showed the encapsulation of a tetraethylammonium ion inside the cage cavity [24]. Moreover, as evidenced above, cages based on L^1^ and L^8^ represent the two limit-cases in terms of ligand flexibility. Hence, in this section, the combined effects of guest absence/presence, guest dimension, and ligands flexibility will be evaluated.

Only the in *vacuum* and in-solvent calculations are considered because the inclusion of dispersion correction on the cages with larger guests is too expensive from the computational point of view. Moreover, the previous section demonstrated that the inclusion of the dispersion contribution has relevant effects on the accuracy of the geometrical parameters but does not revolutionize the distances trends for the helicate/mesocate systems. 

#### 2.2.1. The Guest Role in the Supramolecular Isomerism

**Figure 6:**
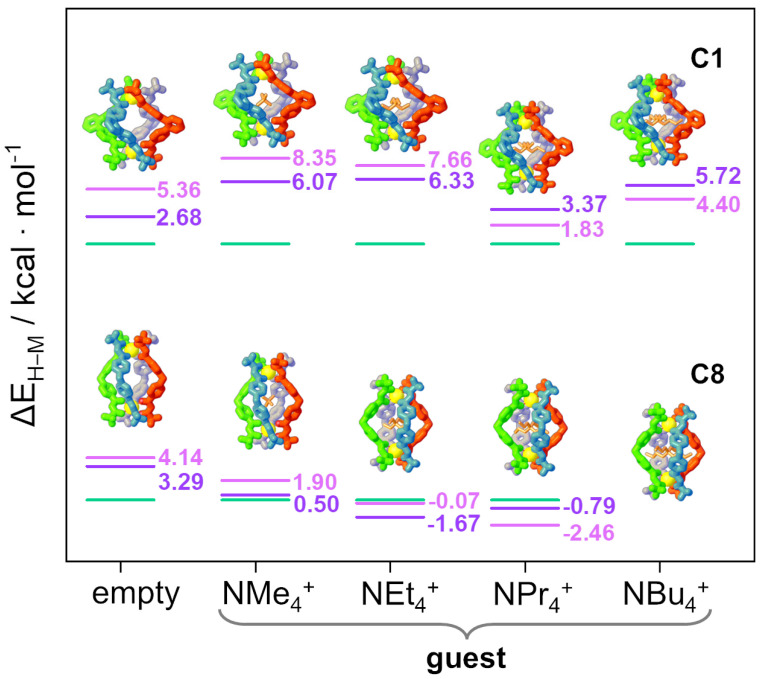
Energy differences ΔE_H–M_ (kcal/mol) between helicate and mesocate isomers. Mesocate (violet in solvent and pink in *vacuum* calculations) and helicate (green). The energy of the helicate is chosen as 0. For the cage NBu_4_⊂**C8**, the helicate did not converge, so ΔE_H–M_ cannot be given.

Considering the most rigid **C1** cage, the absence or the presence of guests with different dimensions has negligible effects on the stabilization of one supramolecular isomer with respect to the other. Indeed, as reported in Figure 6 (top), the most rigid ligand (L^1^) always leads to the helicate isomer, independently of guest presence and its size. Both in *vacuum* (Figure 6, top, pink lines) and in-solvent (Figure 6, top, violet lines) calculations have the same trends for all the considered systems. Expect for the empty cage, the energy differences between in *vacuum* and in-solvent calculations are ca. 2 kcal/mol, hence they are almost negligible from a computational point of view. Guest-induced circular dichroism (CD) and circularly polarized luminescence (CPL) studies confirmed that the empty cage is present in solution as a helicate and SCXRD for the host–guest Eu cage NEt_4_⊂**C1** showed a helicate [24].

The behavior completely changes with the most flexible ligand (L^8^), where the accessible supramolecular isomer is strongly driven by guest presence and dimension. In the empty cage **C8**, or the cage with tetramethylammonium ion NMe_4_⊂**C8**, the helicate resulted the more stable species. Then, increasing the guest dimensions from NEt_4_^+^ to NPr_4_^+^, the mesocate isomer was progressively stabilized (Figure 6, bottom) up to the largest guest NBu_4_^+^, where only the mesocate isomer converged. This seems to suggest a better ability of the mesocate isomer to adapt the cavity size to that of the guest dimensions. Guest-induced CD and CPL studies confirmed that the empty cage is present in solution as a helicate while SCXRD for the host–guest Eu analogue of the NEt_4_⊂**C8** system showed a mesocate. Both the experimental findings are in agreement with the calculations [24]. As observed for the **C1** cages, the in *vacuum* and in-solvent calculations have the same trends. It is important to highlight that, even if small energy variations (below 2 kcal/mol) are not significant, the trends along the guest dimension remain clear, especially with the inclusion of the empty cage. 

#### 2.2.2. The Guest’s Role in the Cage Structure

Geometry optimization of the two isomers (helicate and mesocate) allowed us not only to determine the relative stability of the two species but also to highlight a specific trend in the cage family for structure distortions depending on both the guest dimension (alkylic chain length) and the flexibility of the ligand. The geometrical arrangement of the two ligands, imposed by the scaffold spacers, is different. The angle between the coordinative units is ca. 60° for L^1^ and 110° for L^8^, and hence the **C1** cage is more squashed along the Ln···Ln direction. It is important to note that the tetraalkylammonium ions are allocated on the cages’ equatorial plane (see the green plane in Figure 3A), as evidenced by calculations reported here and previous SCXRD data [24]. It is well-known that guest hosting can induce important variations in host structures. To determine and quantify eventual deformations in these quadruple-stranded hosts, the three distances defined in Figure 3B (d_La–La_, d_opp_, and d_side_) are considered and reported in Table 3. In addition, the area of the equatorial square A_eq_, defined as d_side_^2^, and the inner volume V_inner_, roughly obtained as the volume of the pseudo-octahedron of Figure 3 (V_inner_ = 2/3 ∙ (d_side_)^2^ ∙ d_La__–La_/2 = A_eq_ ∙ d_La__–La_/3), are taken into account (see Table 3).

**Figure 7:**
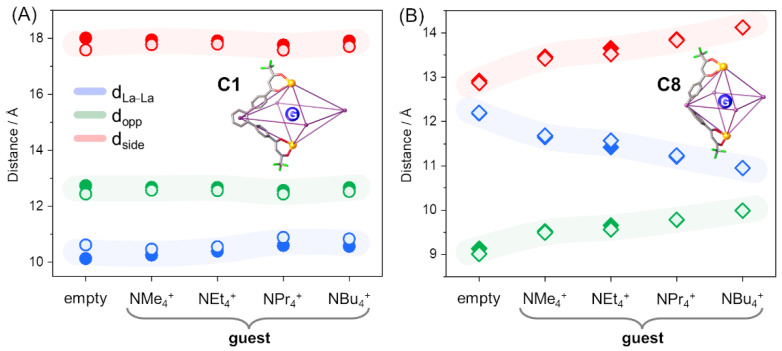
Distance variations (solvent calculations) of the **C1** (**A**) and **C8** (**B**) cages depending on guest presence and size for the helicate (filled symbols) and mesocate (hollow symbols) isomers. Distances are in Å.

In Figure 7A and in Table 3, the variations of the structural parameters for the **C1** cages are reported, moving from the empty to the largest guest. All the calculated distances in solvent show a very tiny variation in dependence of the guest dimension: all parameters have maximum variations below 0.5 Å, and the A_eq_ and V_inner_ with the largest guest increases are 1% and 3%, respectively, with respect to the empty cage for the helicate isomer (Figure 7A, filled circles) as well for the mesocate (see Figure 7A, hollow circles). Hence, **C1** does not undergo structural rearrangement due to guest embedding. On the contrary, large structural variations are obtained with the **C8** series (see Figure 7B and Table 3). These structural modifications are particularly emphasized as the guest size is increased. Indeed, La⋯La distances (d_La–La_) decrease from 12.2 to 10.9 Å going from an empty cage to the NBu_4_⊂**C8** system (see Table 3), while both the parameters related to the equatorial plane increase by about 10%. In particular, the d_side_ distances increase from 9.1 to 10.0 Å and the d_opp_ distances from 12.9 to 14.1 Å. The V_inner_ values enhance around 8% along the series, with an increment over 20% of the A_eq_. As for the **C1** cage, an identical trend is found for both the helicate and mesocate isomers (see Figure 7 and Table 3). 

**C1** and **C8** cages have different V_inner_ values and, considering the empty cages, the **C1** cage is significantly larger, with a volume 40% higher. When considering the A_eq_ parameter, this difference is even larger (51%). This suggests that **C1** cages do not undergo distortions, not only due to the greater rigidity of the ligand, but mainly due to the fact that they already possess a cavity that can easily allocate even large guests. All trends described above for in-solvent calculations are perfectly reproduced both from a qualitative and quantitative point of view also with the lighter in *vacuum* calculations for both cages and both isomers (see Figure S1 and Table S2 of the Supplementary Materials). 

## 3. Materials and Methods

The Amsterdam Density Functional (ADF) program (version 2013.01, Vrije Universities, Theoretical Chemistry, Amsterdam, The Netherlands (2013)) was employed for all calculations [41,42]. The generalized gradient approximation (GGA) PBE exchange–correlation functional [43,44] was used, combined with the TZ2P basis set. The TZ2P is a Slater-type triple-ζ quality basis set augmented with two sets of polarization functions for all the atoms. Moreover, the small frozen-core approximation was employed for core-shell electrons. Core shells up to level 4d for La and 1s for O, C, N, and F were kept frozen. The choice of PBE functional is due to the preview literature on similar systems [23,24,30]. Scalar relativistic effects were considered using the scalar zeroth-order regular approximation (ZORA) [45,46,47]. The numerical integration grid is a refined version of the fuzzy-cell integration scheme developed by Becke. The B3LYP functional [48,49], combined with the TZ2P basis set, was also tested on Eu^3+^ complexes (see complexes in [38]), but there was only a small improvement with respect to the X-ray data, while the enhancement of the computational cost was exponential. Solvent effects were also considered using the COnductor-like Screening MOdel (COSMO) [50] with the default parameters for acetonitrile (dielectric constant ε = 37.5 and a solvent-excluding surface radius of 2.76 Å). Dispersion corrections are included as implemented by Grimme [51] (Grimme3 BJDAMP) for solvent-D calculations. Solvent effects and dispersion corrections are included by reoptimizing the structure. Due to the small energy variations calculated between helicate and mesocate isomers for some ligands, the addition of solvent effects and solvent-with-dispersion effects with a single point calculation is not sufficiently accurate. Indeed, tests on selected systems show energy variations between single-point and re-optimized calculations larger than 2.5 kcal/mol, hence comparable with the helicate/mesocate isomer differences.

## 4. Conclusions

The relative stability of a structure with respect to other accessible systems, and its structural properties, are crucial aspects for MSAs. Thus, the capability to model these parameters and to rationalize the factors that can influence the assembly is mandatory to drive their performances and applications. In this study, the attention was focused on a family of Ln-containing quadruple-stranded helicates/mesocates [La_2_L_4_]^2−^ (**C1**–**C8**) self-assembled with bis-β-diketone ligands. These systems also possess a cavity able to host suitable guests such as NR_4_^+^ cations. The supramolecular isomerism helicate/mesocate was computationally studied according to three different factors: (i) the ligand rigidity and steric hindrance; (ii) the presence of a guest inside the cavity; and (iii) the guest dimension. In particular, the accuracy of the quantum mechanical calculations (in *vacuum*, with the inclusion of the solvent, and with the inclusion of the solvent and the dispersion correction) was taken into account. 

The direct comparison with SCXRD experimental data for NEt_4_⊂**C1** cage to set up the computational protocol shows that all calculations (in *vacuum*, solvent, and solvent-D) are able to reproduce the larger stability of the helicate isomer with respect to the mesocate isomer. Indeed, the difference between the three calculation set-ups is evident in the geometrical parameters, where the inclusion of the dispersion correction reduces the average percentage errors from 4 to 0.3%. The rigidity and the steric hinderance of the ligand scaffolds influence the relative stability of the two isomers: the less rigid and less bulky the ligand, the less stable is the helicate isomer. Indeed, for the most flexible ligand (L^8^), the mesocate becomes the more stable isomer. 

A more detailed analysis of the outcomes for all calculations suggests that:

(i) if one is interested in estimating the stability of one isomer with respect to the others, in *vacuum* calculations are adequate;
(ii) if one is interested in good agreement with geometrical parameters (error on distances <0.5%), the inclusion of solvent and dispersion correction is imperative;
(iii) the accordance with experimental geometrical parameters depends on the flexibility of the ligands: the more flexible the ligand, the larger the average percentage errors.

In addition to the rigidity and the steric hinderance of the ligand, the presence of a guest with a different size strongly influences the relative stability of the two isomers and the MSA structure. Its importance is particularly evident for the most flexible ligand (L^8^), where the helicate is the most stable isomer for the empty and smallest guest, while the mesocate becomes the most stable with larger guests. Since all trends described for the solvent calculations are also perfectly reproduced for the in *vacuum* calculations for both cages and both isomers, if one is interested in the geometrical variation trends in dependence on the size/shape of the guest, it is not necessary to include a solvent effect, which has a higher computational cost. 

## Figures and Tables

**Table 1:** Experimental and calculated values for NEt_4_⊂**C** cages (**C1**, **C3**, **C8**) with the NEt_4_^+^ guest. The **C1** and **C3** cages’ experimental and calculated values are reported for helicate isomer [23,24], while for the **C8** the mesocate isomer values are reported [24]. ΔE_H–M_ is the difference between helicate and mesocate in kcal/mol. A positive value means a more stable helicate with respect to the mesocate, while a negative value denotes the contrary. All distances are in Å. In parenthesis in italics the percentage error between the experimental and calculated distance values.

	In *Vacuum*	Solvent	Solvent-D	SCXRD
NEt_4_⊂**C1**				
Most stable	Helicate	Helicate	Helicate	Helicate
ΔE_H–M_	7.66	6.33	11.55	//
d_La–La_	10.424 (*−0.02*%)	10.392 (*−0.32*%)	10.465 (*0.37*%)	10.426
d_side_	12.682 (*3.63*%)	12.691 (*3.70*%)	12.298 (*0.49*%)	12.238
d_opp_	17.936 (*3.26*%)	17.919 (*3.16*%)	17.390 (*0.12*%)	17.370
NEt_4_⊂**C3**				
Most stable	Helicate	Helicate	Helicate	Helicate
ΔE_H–M_	8.89	5.77	6.12	//
d_La–La_	11.664 (*−3.24*%)	11.610 (*−3.68*%)	11.902 (−*1.26*%)	12.054
d_side_	9.266 (*3.50*%)	9.295 (*3.82*%)	8.952 (*−0.01*%*)*	8.953
d_opp_	13.120 (*3.88*%)	13.142 (*4.05*%)	12.647 (*0.13*%)	12.630
NEt_4_⊂**C8**				
Most stable	Mesocate	Mesocate	Helicate	Mesocate
ΔE_H–M_	−0.07	−1.67	1.77	//
d_La–La_	11.621 (*−1.46*%)	11.570 (*−1.91*%)	11.937 (*1.20*%)	11.795
d_side_	9.568 (*4.65*%)	9.562 (*4.58*%)	9.233 (*0.98*%)	9.143
d_opp_	13.532 (*4.66*%)	13.522 (*4.58*%)	13.057 (*0.98*%)	12.930

**Table 2:** Calculated values for the relative stability ΔE_H–M_ of helicate/mesocate isomers for NEt_4_⊂**C** cages (**C1**–**C8**) are reported. ΔE_H–M_ is the difference between helicate and mesocate in kcal/mol. A positive value means a more stable helicate with respect to the mesocate, while a negative value denotes the contrary.

	In *Vacuum*	Solvent	Solvent-D
NEt_4_⊂**C1**	7.66	6.33	11.55
NEt_4_⊂**C2**	10.10	12.43	13.02
NEt_4_⊂**C3**	8.89	5.77	6.12
NEt_4_⊂**C4**	12.43	10.80	8.57
NEt_4_⊂**C5**	6.49	6.14	6.66
NEt_4_⊂**C6**	7.11	4.81	5.12
NEt_4_⊂**C7**	2.58	1.47	2.37
NEt_4_⊂**C8**	−0.07	−1.67	1.77

**Table 3:** Geometrical parameters and energy differences ΔE_H–M_ between helicate and mesocate isomer for calculations with solvent. Distances are in Å, areas are in Å^2^, volumes are in Å^3^, while ΔE_H–M_ are in kcal/mol. Mesocate values are reported in parentheses. A ΔE_H–M_ negative value means that the mesocate is the most stable isomer.

	Empty	NMe_4_^+^	NEt_4_^+^	NPr_4_^+^	NBu_4_^+^
NR_4_⊂**C1**					
Most stable	Helicate	Helicate	Helicate	Helicate	Helicate
ΔE_H__–__M_	2.68	6.07	6.33	3.37	5.72
d_La__–__La_	10.130 (10.616)	10.253 (10.465)	10.392 (10.552)	10.597 (10.884)	10.564 (10.832)
d_side_	12.736 (12.435)	12.672 (12.570)	12.671 (12.558)	12.565 (12.435)	12.663 (12.524)
d_opp_	18.010 (17.589)	17.943 (17.777)	17.912 (17.759)	17.767 (17.580)	17.907 (17.709)
A_eq_	162 (155)	161 (158)	161 (158)	158 (155)	160 (157)
V_inner_	548 (547)	549 (551)	556 (555)	557 (561)	565 (566)
NR_4_⊂**C8**					
Most stable	Helicate	Helicate	Mesocate	Mesocate	Mesocate
ΔE_H__–__M_	3.29	0.50	−1.67	−0.79	// ^a^
d_La__–__La_	12.187 (12.197)	11.651 (11.682)	11.421 (11.570)	11.208 (11.232)	(10.949)
d_side_	9.133 (9.099)	9.520 (9.492)	9.657 (9.562)	9.779 (9.784)	(9.988)
d_opp_	12.917 (12.868)	13.467 (13.424)	13.658 (13.522)	13.856 (13.834)	(14.125)
A_eq_	83 (83)	91 (90)	93 (91)	96 (96)	(100)
V_inner_	337 (337)	352 (351)	355 (353)	357 (358)	(364)

^a^ The helicate does not converge, so it is not possible to obtain a value of ΔE_H–M_, and only the geometrical parameters for the mesocate are reported.

## Data Availability

Not applicable.

## Supplementary Materials

The following supporting information can be downloaded at: https://www.mdpi.com/article/10.3390/ijms231810619/s1.
