# Supplementary material for: Helicate versus Mesocate in Quadruple-Stranded Lanthanide Cages: A Computational Insight
Source: Int J Mol Sci. 2022 Sep 13;23(18):10619. doi: 10.3390/ijms231810619 (PMC9504305; doi:10.3390/ijms231810619)

International Journal of *Molecular Sciences*

MDPI

Helicate versus mesocate in quadruple-stranded lanthanide cages: a computational insight

Silvia Carlotto[1,2]*, Lidia Armelao[1,3] and Marzio Rancan[2,*]

1 Department of Chemical Sciences (DiSC), University of Padova, via F. Marzolo 1, 35131 Padova, Italy

2 Institute of Condensed Matter Chemistry and Technologies for Energy (ICMATE), National Research Council (CNR), c/o Department of Chemical Sciences (DiSC), University of Padova, via F. Marzolo 1, 35131 Padova, Italy
3 Department of Chemical Sciences and Materials Technologies (DSCTM), National Research Council (CNR), Piazzale A. Moro 7, 00185 Roma, Italy
* Correspondence: silvia.carlotto@unipd.it (S.C.); marzio.rancan@cnr.it or marzio.rancan@unipd.it (M.R.)

Table S1. Calculated values for NEt4⊂**C** cages (**C1** - **C8**) with the NEt_4^+ guest. $\Delta E_{H\text{-}M}$ is the difference between helicate and mesocate in kcal/mol. A positive value means a more stable helicate with respect to the mesocate, while a negative value the contrary. All distances are in Å.

	**in *vacuum***	**solvent**	**solvent-D**
NEt_4⊂**C1**			
Most stable	Helicate	Helicate	Helicate
$\Delta E_{H\text{-}M}$	7.66	6.33	11.55
$d_{La\text{-}La}$	10.424	10.392	10.465
d_{side}	12.682	12.691	12.298
d_{opp}	17.936	17.919	17.390
NEt_4⊂**C2**			
Most stable	Helicate	Helicate	Helicate
$\Delta E_{H\text{-}M}$	10.10	12.43	13.02
$d_{La\text{-}La}$	11.689	11.620	11.946
d_{side}	9.198	9.192	8.818
d_{opp}	12.983	12.964	12.339
NEt_4⊂**C3**			
Most stable	Helicate	Helicate	Helicate
$\Delta E_{H\text{-}M}$	8.89	5.77	6.12
$d_{La\text{-}La}$	11.664	11.610	11.902
d_{side}	9.266	9.295	8.952
d_{opp}	13.120	13.142	12.647
NEt_4⊂**C4**			
Most stable	Helicate	Helicate	Helicate
$\Delta E_{H\text{-}M}$	12.43	10.80	8.57
$d_{La\text{-}La}$	11.625	11.640	12.079
d_{side}	9.324	9.250	8.646
d_{opp}	13.184	13.071	12.113
NEt_4⊂**C5**			
Most stable	Helicate	Helicate	Helicate
$\Delta E_{H\text{-}M}$	6.49	6.14	6.66

d_{La-La}	11.602	11.542	11.410
d_{side}	10.075	10.079	9.972
d_{opp}	14.249	14.254	14.098
NEt_4⊂**C6**			
Most stable	Helicate	Helicate	Helicate
ΔE_{H-M}	7.11	4.81	5.12
d_{La-La}	11.680	11.656	11.887
d_{side}	9.196	9.172	8.909
d_{opp}	13.006	12.972	12.597
NEt_4⊂**C7**			
Most stable	Helicate	Helicate	Helicate
ΔE_{H-M}	2.58	1.47	2.37
d_{La-La}	11.865	11.935	12.262
d_{side}	9.102	8.968	8.550
d_{opp}	12.860	12.665	12.026
NEt_4⊂**C8**			
Most stable	Mesocate	Mesocate	Helicate
ΔE_{H-M}	-0.07	-1.67	1.77
d_{La-La}	11.621	11.570	11.937
d_{side}	9.568	9.562	9.233
d_{opp}	13.532	13.522	13.057

Table S2. Geometrical parameters and energy differences ΔE_{H-M} between helicate and mesocate isomer for calculations with solvent. Distances are in Å, areas are in Å^2, volumes are in Å^3, while ΔE_{H-M} is in kcal/mol. Mesocate values are reported in parenthesis. A ΔE_{H-M} negative value means that the mesocate is the stabler isomer.

	**empty**	**NMe_4^+**	**NEt_4^+**	**NPr_4^+**	**NBu_4^+**
NR_4⊂**C1**					
Most stable	Helicate	Helicate	Helicate	Helicate	Helicate
ΔE_{H-M}	5.36	8.35	7.66	1.83	4.40
d_{La-La}	10.410(10.926)	10.239(10.459)	10.424(10.572)	10.623(10.929)	10.589(10.870)
d_{side}	12.657(12.328)	12.678(12.594)	12.682(12.573)	12.565(12.430)	12.670(12.514)
d_{opp}	17.899(17.436)	17.957(17.810)	17.936(17.782)	17.767(17.576)	17.917(17.696)
A_{eq}	160(152)	161(159)	161(158)	158(155)	161(157)
V_{inner}	556(554)	549(553)	559(557)	559(563)	567(567)
NR_4⊂**C8**					
Most stable	Helicate	Helicate	Mesocate	Mesocate	Mesocate
ΔE_{H-M}	4.14	1.90	-0.07	-2.46	//[a]
d_{La-La}	12.400(12.427)	11.512(11.589)	11.395(11.621)	11.190(11.227)	(10.967)
d_{side}	9.048(8.999)	9.629(9.569)	9.697(9.568)	9.848(9.806)	(10.002)
d_{opp}	12.798(12.726)	13.623(13.532)	13.713(13.532)	13.924(13.865)	(14.145)
A_{eq}	82(81)	93(92)	94(92)	97(96)	(100)
V_{inner}	338(335)	356(354)	357(355)	362(360)	(366)

[a]The helicate does not converge, so it is not possible to obtain a value of ΔE_{H-M}, only the geometrical parameters for the mesocate are reported.

Figure S1. Distance variations (in *vacuum* calculations) of the **C1** (A) and **C8** (B) cages depending on guest presence and size for the helicate (filled symbols) and mesocate (hollow symbols) isomers. Distances are in Å.

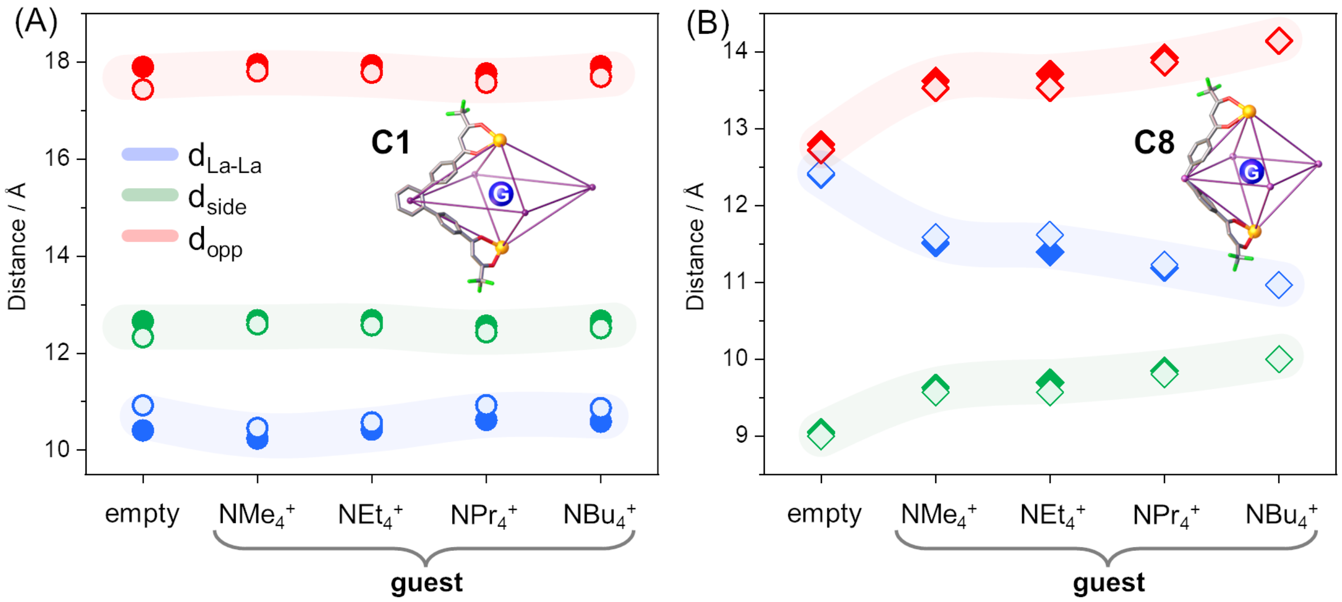

Supplement: Supplementary file 1 [file ijms-23-10619-s001.zip › ijms-1914617-supplementary.pdf]
